# Cognitive behavioral therapy combined with aripiprazole in the treatment of schizophrenia: effects on cognitive function and psychological state

**DOI:** 10.3389/fpsyt.2026.1777230

**Published:** 2026-03-03

**Authors:** Neng Dong, Lili Jiang, Dehui Guo, Jiang Dai, Huicong Jiao, Yiying Wang

**Affiliations:** Department of Psychiatry, Liupanshui Shuikuang Hospital, Liupanshui, Guizhou, China

**Keywords:** aripiprazole, clinical symptoms, cognitive behavioral therapy, cognitive function, emotional state, quality of life, schizophrenia

## Abstract

**Objective:**

The aim of this study is to assess the effects of cognitive behavioral therapy (CBT) combined with aripiprazole on clinical symptoms, cognitive function, and emotional state in patients with schizophrenia.

**Methods:**

A single-center retrospective controlled design was used, with data from the electronic medical records of the psychiatry department of Liupanshui Shuikuang hospital. A total of 168 patients were included and divided into two groups based on whether they received structured CBT intervention: the combined treatment group (aripiprazole + CBT) and the monotherapy group (aripiprazole only), with 84 patients in each group. Propensity score matching was used to reduce bias, and the clinical symptoms, cognitive function, and emotional state of patients were evaluated at baseline (T0), 3 months (T1), and 6 months (T2).

**Results:**

The combined treatment group showed significant improvements in Positive and Negative Syndrome Scale (PANSS) total score, Montreal Cognitive Assessment (MoCA) score, Patient Health Questionnaire-9 (PHQ-9), and Generalized Anxiety Disorder 7 (GAD-7) compared to the monotherapy group (P < 0.05). In terms of quality of life, the combined treatment group showed significantly greater improvement in Schizophrenia Quality of Life Scale (SQLS) total score compared to the monotherapy group (P = 0.006). Multivariate regression analysis revealed that CBT combined treatment significantly enhanced the improvement in PANSS total score and MoCA score, and was associated with reductions in depression and anxiety symptoms. An increase in treatment frequency and intensity further enhanced efficacy. Subgroup analysis showed better treatment responses in younger patients and those with a shorter disease duration.

**Conclusion:**

CBT combined with aripiprazole significantly improves the psychiatric symptoms, cognitive function, and emotional state of patients with schizophrenia, and the frequency and intensity of treatment have a positive impact on efficacy.

## 1 Introduction

Schizophrenia is a chronic and severe mental disorder, characterized mainly by psychiatric symptoms, cognitive impairments, and deficits in emotional and social functioning. Although antipsychotic drugs are significantly effective in alleviating positive symptoms, their effectiveness in improving negative symptoms, cognitive function, and emotional symptoms is limited (1). Therefore, monotherapy often fails to comprehensively improve the overall health condition of patients with schizophrenia, particularly in terms of cognitive function and emotional disorders. Cognitive behavioral therapy (CBT) is a widely applied psychological treatment for various mental disorders and has shown significant efficacy, especially in alleviating anxiety, depressive symptoms, and improving cognitive function. Research has shown that CBT can help patients identify and change negative automatic thoughts, enhance emotional regulation skills, and improve psychological health and social functioning through cognitive restructuring, emotional regulation, and exposure therapy (2, 3). In recent years, CBT has gradually gained attention in the treatment of schizophrenia, especially as an adjunct to pharmacotherapy, showing potential in improving cognitive function and emotional health (4). Although some studies have demonstrated the significant effects of CBT in improving emotional symptoms and cognitive function in patients with schizophrenia (5, 6), most existing studies focus on the effects of CBT as an adjunctive treatment, with insufficient exploration of treatment intensity and differences in treatment responses across different patient groups. Moreover, although aripiprazole has a significant effect on alleviating positive symptoms, its effect on improving negative symptoms and cognitive function is limited (7). These factors result in incomplete improvement in the overall treatment effect for patients (6). Therefore, combining CBT with aripiprazole may be more effective in improving the overall symptoms, especially in cognitive function and emotional health. The study aims to assess the effects of CBT combined with aripiprazole on clinical symptoms, cognitive function, and emotional state in patients with schizophrenia, comparing the efficacy of the combined treatment group and the monotherapy group, and exploring the effects of treatment intensity and patient subgroup responses on treatment outcomes. The results will provide new evidence for comprehensive treatment strategies for schizophrenia and offer important references for clinical practice.

## 2 Materials and methods

### 2.1 General information

A single-center retrospective controlled study design was employed, with data obtained from the electronic medical records of the psychiatry department of Liupanshui Shuikuang hospital. A total of 168 patients who received standardized treatment with aripiprazole and completed baseline and follow-up assessments from May 2023 to June 2025 were included. Patients were naturally divided into the combined treatment group (aripiprazole + CBT) and the monotherapy group (aripiprazole only), with 84 patients in each group. To minimize selection bias, all eligible cases were consecutively included. To reduce baseline bias related to the retrospective and non-randomized design, propensity score matching (PSM) was performed (8). Receipt of combination therapy was treated as the dependent variable, and a logistic regression model was used to estimate the propensity score for each patient. Based on clinical relevance and potential confounding effects, the following baseline covariates were included: age, sex, disease duration, baseline PANSS total score, PANSS positive subscale score, PANSS negative subscale score, PANSS general psychopathology subscale score, and baseline antipsychotic dosage. A 1:1 nearest-neighbor matching method without replacement was applied. The caliper width was set at 0.2 of the standard deviation of the logit of the propensity score. After matching, balance between the two groups was assessed using standardized mean differences (SMD). An SMD value < 0.1 was considered indicative of adequate balance for both continuous and categorical variables. The matched cohort was used for subsequent efficacy analyses.

The baseline time point (T0) was defined as the most recent complete evaluation before the start of stable aripiprazole treatment. Follow-up data was collected at 3 months (T1) and 6 months (T2) to assess changes in clinical symptoms, cognitive function, and emotional state over time. The data used were from previously stored records without additional interventions.

### 2.2 Study subjects

**Figure 1:**
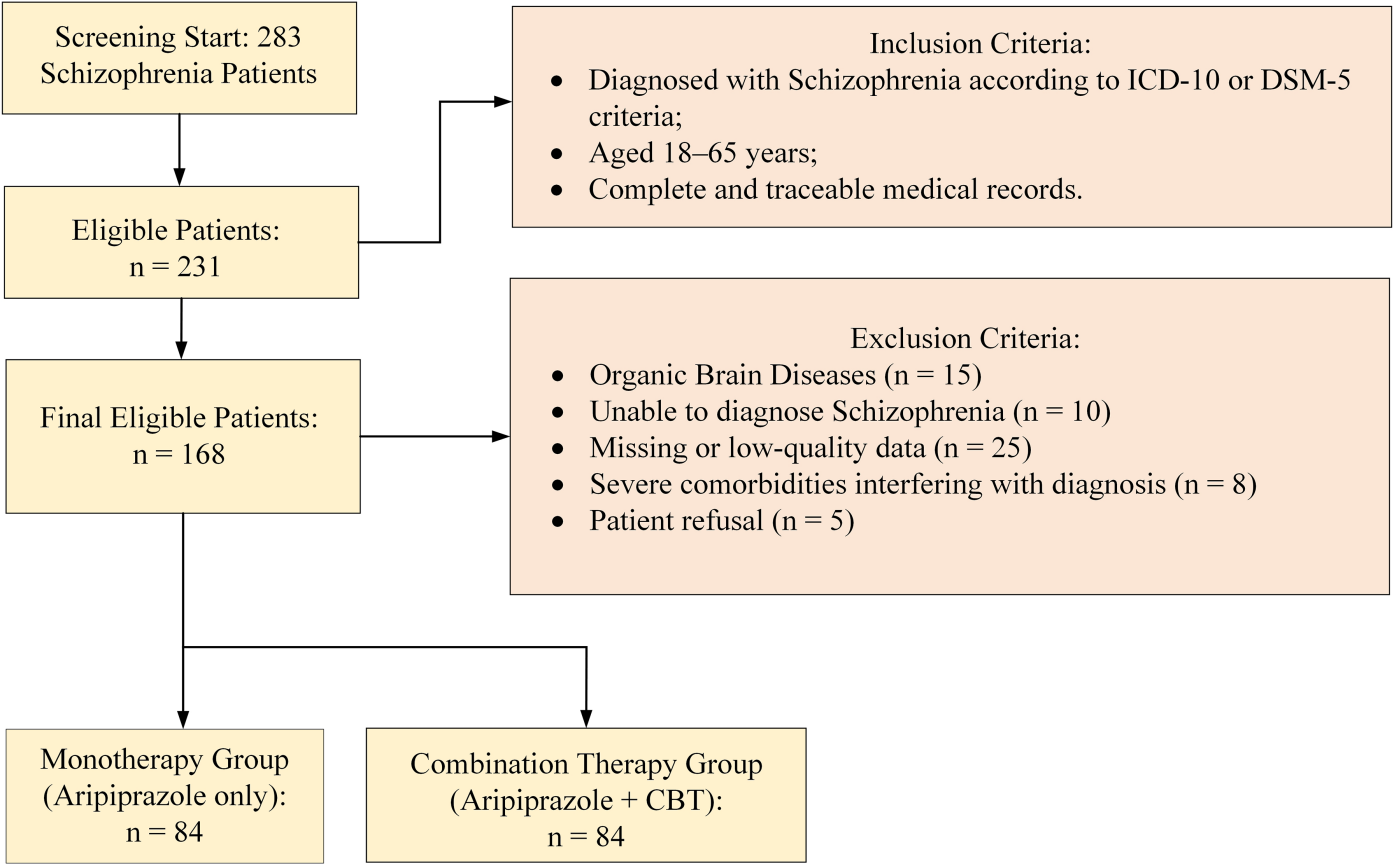
Case screening process.

The study subjects were consecutively enrolled schizophrenia patients, with all cases retrieved from the electronic medical record system. Since this was a retrospective analysis, the study subjects were based on historical medical record data and no additional interventions were applied. The Ethics Committee of Shuikuang Hospital approved this study (Ethics Approval No. skyy-II-20251209), which did not require patients to sign informed consent personally. However, the personal information of all patients was kept strictly confidential and processed in a de-identified manner to ensure compliance with data protection regulations. The inclusion criteria were: (1) meeting the schizophrenia diagnostic criteria in the Diagnostic and Statistical Manual of Mental Disorders, 5th edition (DSM-5) (1), as clearly recorded by a psychiatrist in the medical records; (2) aged 18–65 years; (3) complete and traceable electronic medical record data. The exclusion criteria were: (1) clear organic brain diseases, such as post-traumatic syndrome, brain tumors, epileptic encephalopathy, or severe cerebrovascular lesions; (2) previously diagnosed neurodegenerative diseases, such as Alzheimer’s disease or frontotemporal dementia; (3) significant alcohol or drug dependence (except smoking or occasional drinking); (4) severe physical diseases (such as cardiovascular diseases, diabetes, liver or kidney dysfunction) that could interfere with the assessment of cognitive, emotional, and treatment outcomes; (5) use of other psychiatric medications (such as antidepressants or other antipsychotics) during treatment; (6) missing key variables or poor quality of scale data in the medical records; (7) previous mild cognitive impairment or other psychiatric comorbidities (such as severe depression or anxiety disorders). To ensure the representativeness and reproducibility of the sample, eligible cases were included in the analysis sequentially. The case screening process is shown in **Figure 1**.

### 2.3 Treatment methods

Monotherapy Group (Aripiprazole only): Patients in this group only received aripiprazole (Shanghai Shiyuan Pharmaceutical Co., Ltd.; National Drug Standard H20041506; 5mg tablets). The starting dose was set at 10 mg per day, once daily, regardless of meals. If patients tolerated the drug well and did not show significant clinical improvement, the dose was gradually increased to 15–30 mg/day after 2 weeks, with a maximum dose of 30 mg/day, and this dose was maintained until the end of the study. During maintenance treatment, the treating physician decided whether to continue within the 15–30 mg dose range based on the patient’s clinical response and tolerance. This dose-time design follows the recommended dosing regimen for aripiprazole in the treatment of schizophrenia (1). During this period, patients did not receive any form of CBT or other psychological interventions, and the treatment solely relied on the pharmacological effects of aripiprazole. Patients were clinically followed up monthly to assess drug efficacy and side effects, and dose adjustments were made when necessary.

Combined Treatment Group (Aripiprazole + CBT intervention): In addition to monotherapy, patients in this group also received structured CBT. Cognitive behavioral therapy was delivered by psychiatrists or psychotherapists who had received formal CBT training and had relevant clinical experience. All therapists held a master’s degree or higher in psychology and had at least two years of clinical practice. Before study initiation, all therapists completed a unified training program on the treatment protocol. Interventions were conducted according to a standardized CBT manual. Core components included cognitive restructuring, emotional regulation, and behavioral interventions. These measures were used to ensure consistency in treatment procedures and key session elements. During the study period, therapists completed standardized treatment records after each CBT session. The research team held case review meetings every two weeks. During these meetings, a random sample of cases was selected for review of treatment records. The implementation of core intervention modules and adherence to the predefined treatment protocol were evaluated. When deviations were identified, feedback and protocol calibration were provided during the discussions to minimize inter-therapist variability. The treatment included the following four parts:

1. Emotion Regulation Training: Helped patients identify emotional triggers and learn emotion regulation techniques (e.g., relaxation exercises, meditation). This phase lasted 4 weeks, with one session per week, each lasting 50 minutes;
2. Cognitive Restructuring: Helped patients identify and change negative automatic thoughts, and learn to think more positively and rationally. This phase lasted 8 weeks, with one session per week, each lasting 50 minutes;
3. Behavioral Correction: Helped patients identify and change maladaptive behavior patterns using gradual exposure and behavior reinforcement techniques. This phase lasted 8 weeks, with one session per week, each lasting 50 minutes;
4. Stress Management and Coping Strategies: Helped patients identify sources of stress in their lives and learn coping strategies (e.g., time management, social support utilization). This phase lasted 6 weeks, with one session per week, each lasting 50 minutes.

### 2.4 Observation indicators

All scales and data used in this study were derived from the electronic medical record system. Baseline and follow-up data for all patients were recorded and rated by trained physicians and psychological evaluators according to standard procedures. The details are as follows:

1. Disease Severity: Assessed according to the subtype of schizophrenia (positive, negative, mixed), first episode or recurrent status, previous hospitalization frequency, history of suicidal or aggressive behavior, and baseline Positive and Negative Syndrome Scale (PANSS) (positive, negative, and general psychopathology subscales) (9). The PANSS total score ranges from 30 to 210, with higher scores indicating more severe psychiatric symptoms.
2. Cognitive Function: Measured using the Montreal Cognitive Assessment (MoCA) (10), with a total score ranging from 0 to 30, with higher scores indicating better cognitive function.
3. Psychological State and Intervention Information: Psychological state was assessed using the Patient Health Questionnaire-9 (PHQ-9) (11) and the Generalized Anxiety Disorder 7 (GAD-7) (12), which reflect depression and anxiety levels. The PHQ-9 total score ranges from 0 to 27, and the GAD-7 ranges from 0 to 21, with higher scores indicating more severe symptoms. Quality of life was assessed using the Schizophrenia Quality of Life Scale (SQLS), which includes three dimensions: motivation and vitality (0-30), somatic-psychological (0-30), and symptom discomfort (0-40), with a total score ranging from 0 to 100, with higher scores indicating worse quality of life.
4. Treatment-Related Data: Includes the starting and maintenance doses of aripiprazole, the method of administration (oral or long-acting injection), the use of other antipsychotic medications or mood stabilizers, common side effects (e.g., weight changes, metabolic abnormalities, and extrapyramidal symptoms), and relapse or rehospitalization events within 6 months of treatment. CBT-related information includes whether the patient received structured CBT intervention and the number of sessions, which reflects adherence and treatment intensity. All scales were completed by trained physicians or psychological evaluators following standardized procedures.

### 2.5 Dose-response analysis

To assess the impact of different treatment intensities on efficacy, the study performed a dose-response analysis using the actual maintenance doses of aripiprazole and CBT treatment frequencies as one of the main independent variables. Treatment frequencies were divided into low frequency (once a week), medium frequency (twice a week), and high frequency (three times a week). The doses of aripiprazole were classified as low dose (10–20 mg/day), standard dose (20–30 mg/day), and high dose (30 mg/day). The study analyzed changes in PANSS scores, MoCA scores, PHQ-9, and GAD-7 scores as dependent variables using linear regression models to explore the relationship between treatment intensity and treatment outcomes.

### 2.6 Moderator analysis and subgroup analysis

To explore how baseline characteristics of patients influenced treatment outcomes, the study divided patients into subgroups based on age, disease duration, cognitive level (MoCA score), and anxiety/depression levels (PHQ-9, GAD-7 scores). Specifically, the groups were divided as follows: younger group (<40 years), middle-aged group (40–60 years), older group (>60 years); early disease duration group (<5 years) and late disease duration group (>5 years); as well as different cognitive abilities and emotional levels (grouped based on median MoCA, PHQ-9, and GAD-7 scores). Multivariate regression analysis was then used to examine whether these baseline characteristics moderated the treatment outcomes, particularly the differences in PANSS total score, MoCA improvement, and emotional symptom ratings.

### 2.7 Statistical analysis

All data were analyzed using SPSS. First, for data that did not meet normal distribution, the Mann–Whitney U test was used for non-parametric comparisons. Continuous variables were expressed as mean ± standard deviation or median (interquartile range) and compared between groups using independent sample t-tests or Mann–Whitney U tests. Categorical variables were expressed as frequencies and percentages, and the χ² test was used for comparisons.

In multivariate regression analysis, in addition to the basic covariates (such as age, sex, disease duration), we also included factors such as lifestyle (e.g., smoking, drinking habits, physical activity), social support (e.g., family support, friend support), education level, and income, to further control for potential confounders. In addition, sensitivity analysis was performed to verify the robustness of the regression model under different combinations of covariates. The results showed that treatment effects remained stable under different covariate conditions, further supporting the effectiveness of CBT.

For missing data, the study used multiple imputation (Multiple Imputation) to generate complete datasets through repeated imputations based on existing data, which reduced potential bias due to missing data. During the imputation process, multiple covariates and dependencies were considered to ensure the accuracy and robustness of the imputed results. The imputed dataset was used for regression analysis to further validate the robustness of the results. All analyses were performed with two-sided tests, and a P value < 0.05 was considered statistically significant.

## 3 Results

### 3.1 Baseline data comparison

After propensity score matching, the two groups showed comparable distributions in baseline demographic and clinical characteristics. These included age, sex composition, disease duration, educational level, living arrangement, smoking status, alcohol consumption, marital status, and major physical comorbidities. With respect to disease severity indicators, good balance was also observed between groups in baseline PANSS total score and in the positive, negative, and general psychopathology subscale scores. All baseline variables demonstrated standardized mean differences below 0.1, indicating satisfactory post-matching comparability between the two groups. (**Supplementary Table 1**).

### 3.2 PANSS improvement results

**Figure 2:**
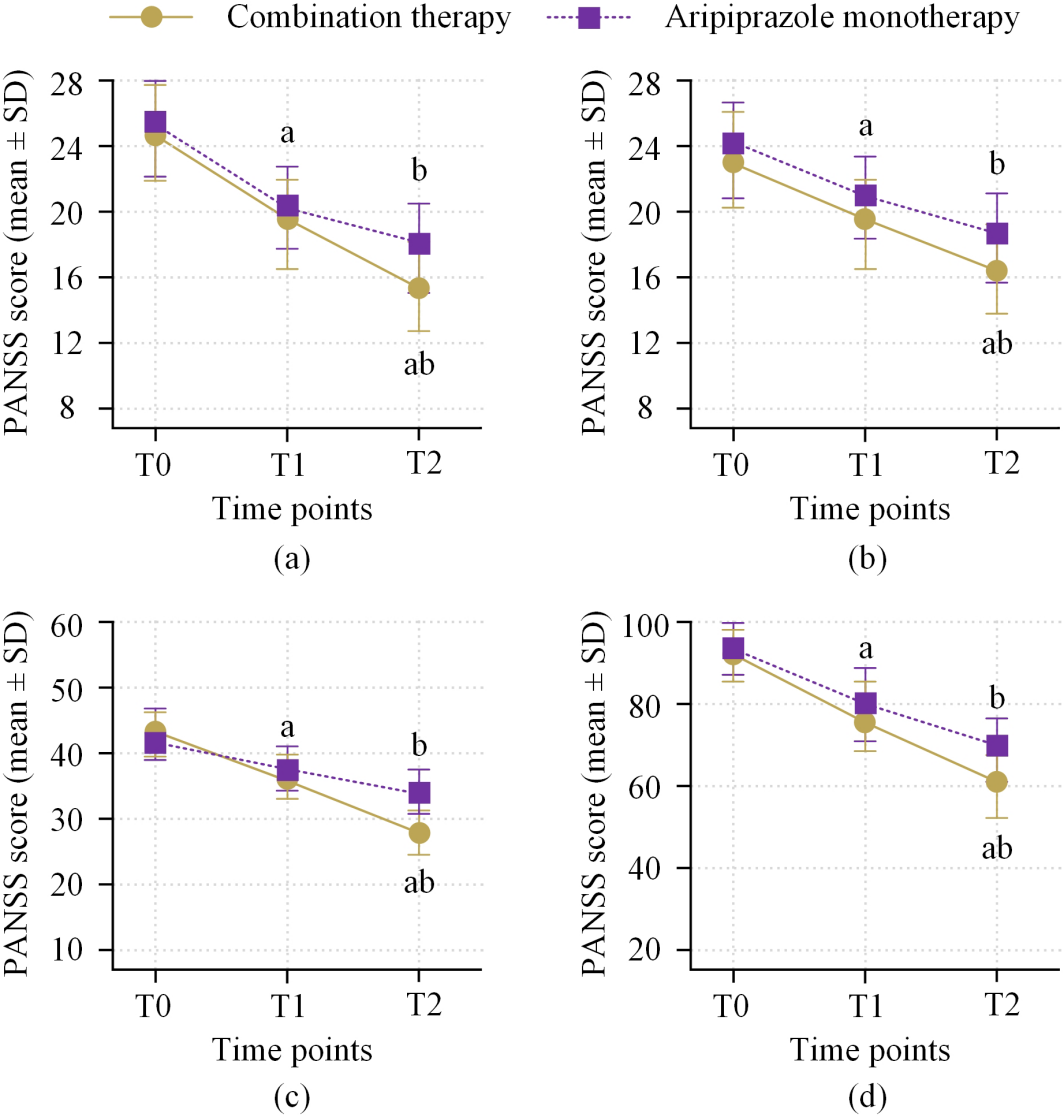
Comparison of PANSS improvement between two groups. **(a)** PANSS positive symptoms; **(b)** PANSS negative symptoms; **(c)** PANSS general psychopathology; **(d)** PANSS total score. Note: a represents a significant decrease in within-group scores comparing T1 with T0 (P < 0.05); b represents a significant decrease in within-group scores comparing T2 with T0 (P < 0.01); ab indicates that at T2, the improvement in the combined treatment group was significantly better than the monotherapy group (P < 0.01).

Compared to T0, both groups showed significant reductions in PANSS total score and subscale scores at T1 and T2 (within-group comparisons, P < 0.05). At the T2 assessment, the combined treatment group had a lower PANSS total score than the monotherapy group, and there was a significant difference in the improvement between the two groups (improvement value of −28.3 ± 9.5 for the combined treatment group, and −21.9 ± 10.1 for the monotherapy group, P = 0.008). The trends for improvement in positive, negative, and general psychopathology subscales were consistent with the total score. At T2, the improvement in all subscales was greater in the combined treatment group compared to the monotherapy group (P < 0.01 for all) (**Figure 2**, **Supplementary Table 2**).

### 3.3 Cognitive function results

Both groups showed significant improvements in MoCA scores at T1 and T2 compared to baseline (within-group comparisons, P < 0.05). At T2, the combined treatment group had a higher MoCA score than the monotherapy group (25.8 ± 3.2 *vs*. 24.0 ± 3.3, P = 0.001). Further comparison of the improvement from T0 to T2 showed that the combined treatment group had a greater improvement in MoCA (4.4 ± 2.6 *vs*. 2.9 ± 2.5, P = 0.002) (**Table 1**).

**Table 1:** MoCA score changes over time (mean ± SD).

Measure	Time point	Combined treatment group (n=84)	Monotherapy group (n=84)	Between-group t-value	Between-group P-value
MoCA Score	T0	21.4 ± 3.5	21.1 ± 3.6	0.54	0.590
—	T1	23.9 ± 3.4^a^	22.8 ± 3.5^a^	2.07	0.040
—	T2	25.8 ± 3.2^b^	24.0 ± 3.3^b^	3.47	0.001
Improvement ΔT1–T0	—	2.8 ± 1.6	2.0 ± 1.5	2.65	0.010
Improvement ΔT2–T0	—	4.4 ± 2.6	2.9 ± 2.5	3.21	0.002

a, Significant improvement in within-group scores comparing T1 with T0 (P < 0.001); b, Significant improvement in within-group scores comparing T2 with T0 (P < 0.001); ΔT1–T0 and ΔT2–T0 represent the improvement in scores from T1 and T2 relative to baseline, respectively.

### 3.4 Emotional symptoms comparison

Depression and anxiety scores in both groups decreased with treatment time (PHQ-9 and GAD-7 within-group comparisons, P < 0.05). At T2, the combined treatment group had lower PHQ-9 scores compared to the monotherapy group (6.2 ± 3.1 *vs*. 8.0 ± 3.4, P = 0.003), and a similar difference was observed in GAD-7 scores (5.8 ± 2.9 *vs*. 7.4 ± 3.2, P = 0.004). When comparing the change from T0 to T2, the combined treatment group showed a larger reduction in depression and anxiety scores than the monotherapy group (PHQ-9: -5.3 ± 2.9 *vs*. -3.3 ± 2.7;GAD-7:-4.8 ± 2.7 *vs*. -3.2 ± 2.6; P values all < 0.01) (**Supplementary Table 3**).

### 3.5 Quality of life comparison

At both T1 and T2, both groups showed a decrease in their SQLS total scores compared to baseline (within-group comparisons, P < 0.05). At T2, the combined treatment group had a significantly lower SQLS total score than the monotherapy group (42.5 ± 11.3 *vs*. 48.7 ± 12.1, P = 0.006). When analyzing the subscales, the combined treatment group also showed lower scores in the motivation and vitality and somatic-psychological dimensions (motivation and vitality: 13.2 ± 4.8 *vs*. 15.6 ± 5.1, P = 0.010; somatic-psychological: 14.0 ± 5.0 *vs*. 16.3 ± 5.4, P = 0.018), while the difference in the symptom discomfort dimension was not significant (P > 0.05) (**Table 2**).

**Table 2:** Changes in SQLS scores over time (mean ± SD).

Measure	Time point	Combined treatment group (n=84)	Monotherapy group (n=84)	Between-group t-value	Between-group P-value
SQLS Total Score	T0	58.6 ± 12.5	58.1 ± 12.8	0.25	0.803
—	T1	49.8 ± 11.8^a^	53.4 ± 12.5^a^	1.82	0.071
—	T2	42.5 ± 11.3^b^	48.7 ± 12.1^b^	2.78	0.006
Improvement ΔT2–T0	—	−16.1 ± 9.4	− 9.4 ± 8.7	3.52	0.001
Motivation and Vitality	T0	18.5 ± 5.7	18.1 ± 5.9	0.46	0.648
—	T1	15.8 ± 5.1^a^	17.2 ± 5.4^a^	1.74	0.085
—	T2	13.2 ± 4.8^b^	15.6 ± 5.1^b^	2.63	0.010
Somatic-Psychological	T0	18.9 ± 6.1	19.1 ± 6.0	0.21	0.832
—	T1	16.7 ± 5.5^a^	18.2 ± 5.7^a^	1.76	0.081
—	T2	14.0 ± 5.0^b^	16.3 ± 5.4^b^	2.39	0.018
Symptom Discomfort	T0	21.2 ± 6.2	20.9 ± 6.5	0.28	0.780
—	T1	19.1 ± 6.0^a^	20.3 ± 6.3^a^	1.27	0.206
—	T2	18.0 ± 5.8^b^	18.9 ± 6.1^b^	1.05	0.296

a, Significant decrease in within-group scores comparing T1 with T0 (P < 0.05); b, Significant decrease in within-group scores comparing T2 with T0 (P < 0.05).

### 3.6 Multiple regression analysis

Changes in PANSS total score and MoCA score were used as dependent variables in a multivariate linear regression model. Whether patients received CBT was included as the main independent variable, and covariates such as age, gender, disease duration, baseline PANSS total score, and comorbidity count were also included. The results showed that CBT combined treatment was significantly associated with greater reductions in PANSS total score and greater improvements in MoCA score (PANSS: β = −6.2, 95% CI −9.1 to −3.3, P < 0.001; MoCA: β = 1.3, 95% CI 0.6 to 2.0, P < 0.001). Further adjustment for baseline depression and anxiety scores confirmed the significance of CBT effects, indicating that CBT independently contributed to improvements in psychiatric symptoms and cognitive function (**Supplementary Table 4**).

### 3.7 Comparison of treatment regimens and treatment effects in patient subgroups

**Figure 3:**
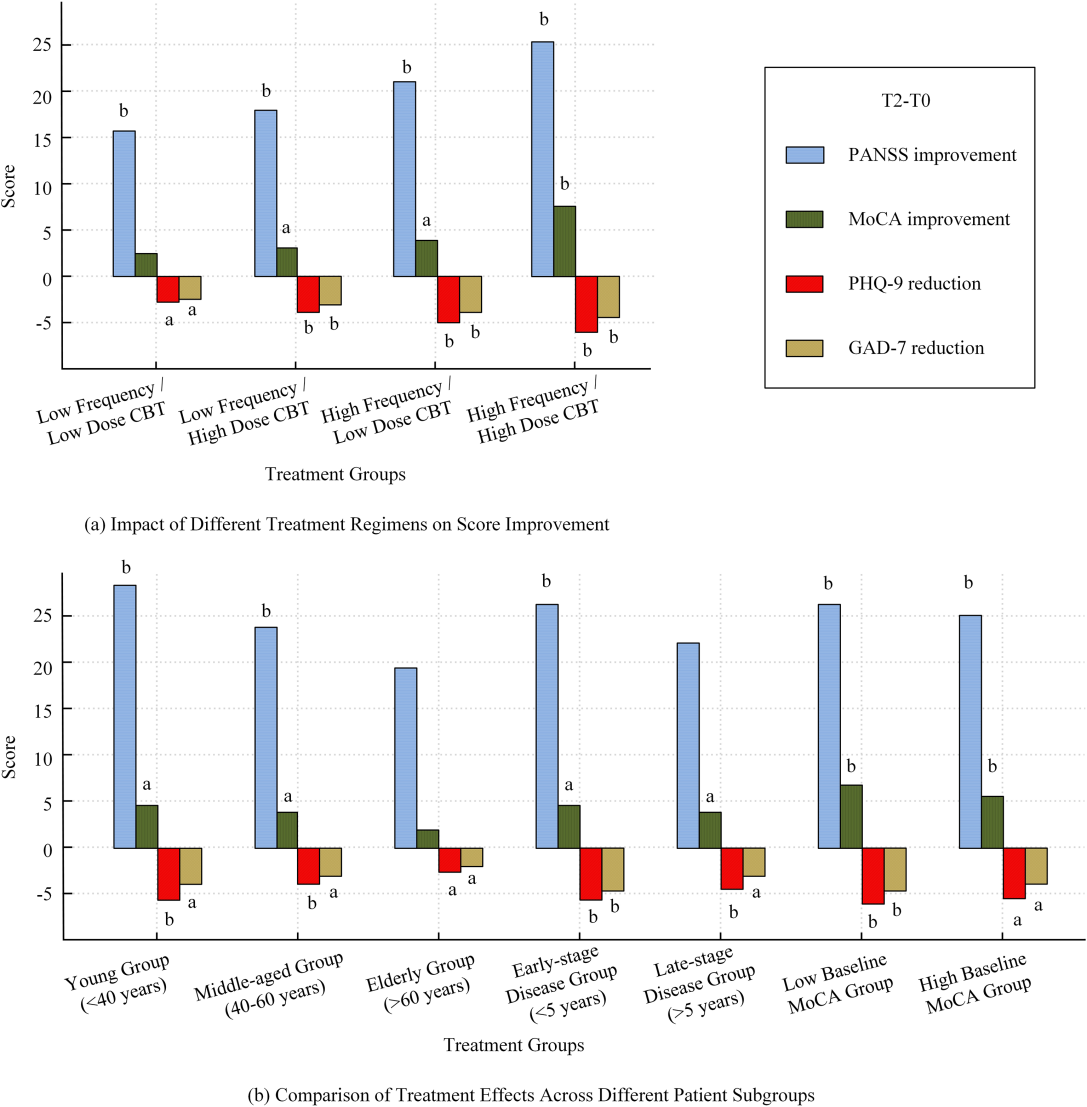
Comparison of treatment effects on score improvement across regimens and patient subgroups; **(a)** Impact of different treatment regimens on score improvement; **(b)** Comparison of treatment effects across different patient subgroups. Note: a, T2 compared with T0, within-group score difference is statistically significant (P < 0.05); b, T2 compared with T0, within-group score difference is statistically significant (P < 0.01).

The study results showed that increased treatment intensity significantly improved patients’ symptoms and cognitive function. The high-dose, high-frequency CBT treatment regimen led to the greatest improvements in PANSS total scores and MoCA scores, with PHQ-9 scores improving by -6.3 points and GAD-7 scores improving by -4.5 points (P < 0.05). In contrast, the low-dose, low-frequency CBT group showed much smaller improvements, with a PANSS total score improvement of 15.6 points (P = 0.002) and a MoCA score improvement of 2.4 points (P = 0.049). This result indicates that both the dosage and frequency of treatment are positively correlated with efficacy (**Figure 3a**).

There were significant differences in treatment effects across different patient subgroups. The younger patient group (<40 years) showed significantly greater improvements in PANSS and MoCA scores compared to the older patient group (>60 years) (P < 0.01). Additionally, patients with early disease duration (<5 years) showed better PANSS improvement (25.8 points *vs*. 21.1 points, P = 0.009), and greater reductions in PHQ-9 and GAD-7 scores (-5.8 points and -4.3 points, respectively, P < 0.05). Patients with low baseline cognitive levels showed significantly better improvement in PANSS, MoCA, PHQ-9, and GAD-7 scores compared to those with high baseline cognitive levels (P < 0.05) (**Figure 3b**).

## 4 Discussion

The study aimed to assess the effects of cognitive behavioral therapy (CBT) combined with aripiprazole on clinical symptoms, cognitive function, and emotional state in patients with schizophrenia. The results showed that, compared with aripiprazole monotherapy, the combined treatment achieved greater improvements in PANSS total score, cognitive performance assessed by the MoCA, and anxiety and depressive symptoms measured by the PHQ-9 and GAD-7. These findings suggest that CBT serves as an effective adjunct to pharmacological treatment and enhances therapeutic outcomes across multiple domains.

With respect to psychiatric symptoms, the combined treatment group showed significantly greater improvements in the PANSS total score and all subscale scores than the monotherapy group. This finding indicates that CBT can further alleviate positive symptoms, negative symptoms, and general psychopathology beyond the effects of medication alone. Aripiprazole, as a partial agonist, helps alleviate positive symptoms by modulating the dopamine system, but its effect on negative symptoms and emotional symptoms is limited (13). Through cognitive restructuring, behavioral activation, and emotional regulation training, CBT helps patients identify and modify negative automatic thought patterns. In this way, CBT may partially compensate for the limitations of pharmacological treatment at the psychological and behavioral levels (14, 15).

Regarding the improvement in cognitive function, the study found that the combined treatment group showed significantly better improvement in MoCA scores compared to the monotherapy group. Cognitive impairment is one of the core symptoms of schizophrenia, and it is usually difficult to achieve significant improvement through monotherapy (6). This study indicates that CBT can significantly improve patients’ cognitive function, particularly in executive function, attention, and memory, which is consistent with existing research (2). CBT helps patients identify and correct maladaptive automatic thoughts, enhancing self-efficacy and thus improving cognitive function (4). In addition, emotional regulation and stress coping training may indirectly improve cognitive function, as anxiety, depression, and other emotional issues often exacerbate cognitive impairments (16). It should be noted that the MoCA is a brief screening tool designed for global cognitive assessment and does not provide detailed evaluation of specific cognitive domains. Therefore, the observed improvements in MoCA scores should be interpreted with caution. Future studies may incorporate more comprehensive neuropsychological batteries to better characterize the cognitive effects of CBT.

In terms of emotional symptoms, the significant reductions in depression and anxiety scores in the combined treatment group suggest that CBT has important value for addressing comorbid emotional disturbances in schizophrenia. Anxiety and depressive symptoms often interact with psychotic symptoms and negatively affect cognitive function and quality of life (17). Anxiety and depression symptoms in schizophrenia patients are often difficult to treat and do not always correspond to the effects of pharmacotherapy. CBT, through cognitive restructuring, emotional regulation, and exposure therapy, can provide a more comprehensive treatment effect (18).

Quality-of-life analyses showed that patients receiving CBT combined with aripiprazole experienced greater overall improvement than those receiving monotherapy. This finding indicates that the addition of CBT may have a positive impact on daily functioning and subjective health perception. Previous studies have shown that improvements in quality of life are closely related to reductions in anxiety and depressive symptoms (19). Through emotional regulation and cognitive restructuring in CBT, patients can better cope with the emotional distress caused by schizophrenia, thereby improving daily functioning and psychological adaptability (20).

From a theoretical perspective, emerging neurobiological and systems-level frameworks provide complementary insights beyond traditional psychological–behavioral mechanisms. Recent studies have suggested that acute psychosis and other psychiatric disorders may be associated with disturbances in brain homeostasis, particularly involving dysfunction of the brain clearance system, known as the glymphatic system (21). Clinical evidence reported by Barlattani et al. demonstrated preliminary signs of glymphatic dysfunction in patients hospitalized for acute psychotic episodes, suggesting a potential role of this system in disease onset and recovery processes (22). Within this framework, CBT may indirectly support brain homeostasis by improving sleep structure, reducing psychological stress, and modulating autonomic function, thereby exerting beneficial effects on cognitive and emotional outcomes. Although the present study did not directly assess neurobiological markers, this perspective provides a useful theoretical background for interpreting the multidimensional benefits observed with combined treatment.

In addition, treatment intensity appeared to play an important role in therapeutic outcomes. Higher-frequency CBT interventions were associated with greater improvements in psychiatric symptoms, cognitive function, and emotional status compared with lower-frequency interventions. This finding supports a positive association between treatment frequency and efficacy (23). More intensive intervention may enhance patient adherence and engagement, allowing concentrated cognitive restructuring and emotional regulation within a shorter time frame, which may strengthen overall treatment effects (24). Subgroup analyses further indicated that younger patients and those with a shorter disease duration showed more pronounced responses to treatment, suggesting that neural plasticity and disease stage may influence intervention effectiveness (25). Future studies incorporating neuroimaging methods may help clarify the mechanisms underlying differential treatment responses across patient subgroups (26).

Despite these positive findings, several limitations should be acknowledged. First, the retrospective design limits the ability to fully exclude selection bias and unmeasured confounding factors. Although PSM was applied to reduce baseline imbalance, inherent limitations of retrospective studies remain. Prospective randomized controlled trials are needed to provide stronger evidence for the efficacy of CBT combined with aripiprazole. Second, the study was conducted at a single center with a relatively small sample size, which may limit the generalizability of the findings. In addition, patients receiving other psychotropic medications were excluded to minimize pharmacological confounding. While this approach allowed clearer evaluation of the combined treatment effect, it may reduce applicability to more complex real-world clinical populations. Future studies in broader patient cohorts are warranted to further assess the effectiveness and generalizability of this treatment strategy.

## 5 Conclusion

In conclusion, this study demonstrates that CBT combined with aripiprazole is associated with significant improvements in psychiatric symptoms, cognitive function, and emotional status in patients with schizophrenia. This combined treatment approach shows clear clinical benefit and offers a broader perspective for understanding treatment response through the integration of psychological–behavioral and neurobiological frameworks.

## Data Availability

The raw data supporting the conclusions of this article will be made available by the authors, without undue reservation.
