# Supplementary material for: Cognitive behavioral therapy combined with aripiprazole in the treatment of schizophrenia: effects on cognitive function and psychological state
Source: Front Psychiatry. 2026 Mar 3;17:1777230. doi: 10.3389/fpsyt.2026.1777230 (PMC12992246; doi:10.3389/fpsyt.2026.1777230)
Supplement: Supplementary file 1 [file Table1.docx]

Supplementary Material

# Supplementary Tables

**Supplementary Table 1. Baseline Data Comparison Between Two Groups**

| Variable | Combined Treatment Group (n=84) | Monotherapy Group (n=84) | Statistic | P-value |
| --- | --- | --- | --- | --- |
| **Age (years)** | 34.8 ± 9.6 | 35.4 ± 10.1 | t = 0.39 | 0.70 |
| **Gender** | — | — | — | — |
| Male | 47 (55.95%) | 49 (58.33%) | χ² = 0.10 | 0.75 |
| Female | 37 (44.05%) | 35 (41.67%) |  |  |
| **Disease Duration** | — | — | — | — |
| Short-term (<5 years) | 40（47.6%） | 42（50%） | χ² = 0.10 | 0.75 |
| Medium-term (5-10 years) | 28（33.3%） | 26（31%） | χ² = 0.42 | 0.67 |
| Long-term (>10 years) | 16（19%） | 16（19%） | χ² = 0.40 | 0.69 |
| **Education Level** | — | — | — | — |
| Low education (<6 years) | 29（34.5%） | 31（36.9%） | χ² = 0.10 | 0.91 |
| Medium education (6-12 years) | 32（38.1%） | 30（35.7%） | χ² = 0.05 | 0.80 |
| High education (>12 years) | 23（27.4%） | 23（27.4%） | χ² = 0.15 | 0.88 |
| **Living Arrangement** | — | — | — | — |
| Living Alone | 15（17.9%） | 14（16.7%） | χ² = 0.10 | 0.75 |
| Living with Family | 69（82.1%） | 70（83.3%） | χ² = 0.05 | 0.80 |
| **Smoking Status** | — | — | — | — |
| Smoker | 37（44.0%） | 39（46.4%） | χ² = 0.10 | 0.80 |
| Non-smoker | 47（56.0%） | 45（53.6%） | χ² = 0.05 | 0.75 |
| **Drinking Status** | — | — | — | — |
| Drink Alcohol | 31（36.9%） | 29（34.5%） | χ² = 0.05 | 0.80 |
| Do Not Drink | 53（63.1%） | 55（65.5%） | χ² = 0.05 | 0.75 |
| **Marital Status** | — | — | — | — |
| Unmarried | 52 (61.90%) | 55 (65.48%) | χ² = 0.23 | 0.63 |
| Married | 32 (38.10%) | 29 (34.52%) |  |  |
| **Physical Comorbidities** | — | — | — | — |
| 0 | 49 (58.33%) | 46 (54.76%) | χ² = 0.29 | 0.86 |
| 1 | 25 (29.76%) | 26 (30.95%) |  |  |
| ≥2 | 10 (11.90%) | 12 (14.29%) |  |  |
| **Family History of Mental Illness** | — | — | — | — |
| Yes | 14 (16.67%) | 12 (14.29%) | χ² = 0.24 | 0.62 |
| No | 70 (83.33%) | 72 (85.71%) |  |  |
| **First Episode** | — | — | — | — |
| Yes | 31 (36.90%) | 29 (34.52%) | χ² = 0.10 | 0.75 |
| No | 53 (63.10%) | 55 (65.48%) |  |  |
| **Previous Hospitalizations** | — | — | — | — |
| 0 | 28 (33.33%) | 30 (35.71%) | χ² = 0.18 | 0.91 |
| 1 | 33 (39.29%) | 31 (36.90%) |  |  |
| ≥2 | 23 (27.38%) | 23 (27.38%) |  |  |
| **PANSS Positive Score** | 24.8 ± 4.7 | 25.2 ± 5.1 | t = 0.53 | 0.60 |
| **PANSS Negative Score** | 23.5 ± 5.0 | 24.1 ± 5.3 | t = 0.72 | 0.47 |
| **PANSS General Psychopathology Score** | 43.2 ± 7.4 | 42.7 ± 7.9 | t = 0.39 | 0.70 |
| **PANSS Total Score** | 91.5 ± 12.6 | 92.0 ± 13.1 | t = 0.25 | 0.80 |

**Supplementary Table 2. Changes in PANSS scores at different time points in the two groups (mean ± SD)**

| Outcome | Time point | Combination therapy group (n = 84) | Monotherapy group (n = 84) | Between-group P value |
| --- | --- | --- | --- | --- |
| **PANSS total Score** | T0 | 92.6 ± 12.4 | 91.8 ± 11.9 | 0.68 |
|  | T1 | 74.2 ± 11.3ᵃ | 78.6 ± 12.1ᵃ | — |
|  | T2 | 64.3 ± 10.2ᵇ | 69.9 ± 10.8ᵇ | 0.008 |
| **PANSS positive symptoms** | T0 | 24.8 ± 5.3 | 24.5 ± 5.1 | 0.74 |
|  | T1 | 18.9 ± 4.6ᵃ | 20.7 ± 4.8ᵃ | — |
|  | T2 | 15.6 ± 4.1ᵇ | 18.2 ± 4.4ᵇ | < 0.01 |
| **PANSS negative Symptoms** | T0 | 23.6 ± 5.0 | 23.9 ± 4.8 | 0.66 |
|  | T1 | 19.6 ± 4.3ᵃ | 21.3 ± 4.5ᵃ | — |
|  | T2 | 16.8 ± 3.9ᵇ | 18.5 ± 4.2ᵇ | < 0.01 |
| **PANSS general psychopathology** | T0 | 44.2 ± 7.1 | 43.4 ± 6.9 | 0.59 |
|  | T1 | 36.9 ± 6.5ᵃ | 39.8 ± 6.8ᵃ | — |
|  | T2 | 28.5 ± 6.1ᵇ | 34.9 ± 6.4ᵇ | < 0.01 |

**Note:** a and b denote significant within-group decreases compared with baseline T0 (P < 0.05 and P < 0.01, respectively).

**Supplementary Table 3. Changes and Improvement of PHQ-9 and GAD-7 Scores Over Time (Mean ± SD)**

| Measure | Time Point | Combined Treatment Group (n=84) | Monotherapy Group (n=84) | Between-group t-value | Between-group P-value |
| --- | --- | --- | --- | --- | --- |
| **PHQ-9 Score** | T0 | 11.6 ± 3.1 | 11.4 ± 3.0 | 0.43 | 0.668 |
| — | T1 | 8.2 ± 2.6ᵃ | 9.5 ± 2.7ᵃ | 2.40 | 0.018 |
| — | T2 | 6.2 ± 3.1ᵇ | 8.0 ± 3.4ᵇ | 3.01 | 0.003 |
| **Improvement ΔT1–T0** | — | −3.4 ± 2.4 | −1.9 ± 2.2 | 3.25 | 0.001 |
| **Improvement ΔT2–T0** | — | −5.3 ± 2.9 | −3.3 ± 2.7 | 3.20 | 0.002 |
| **GAD-7 Score** | **T0** | 10.7 ± 3.0 | 10.9 ± 3.1 | 0.42 | 0.676 |
| — | **T1** | 7.2 ± 2.3ᵃ | 8.3 ± 2.6ᵃ | 2.18 | 0.031 |
| — | **T2** | 5.8 ± 2.9ᵇ | 7.4 ± 3.2ᵇ | 2.67 | 0.004 |
| **Improvement ΔT1–T0** | — | −3.5 ± 2.4 | −2.6 ± 2.3 | 2.05 | 0.042 |
| **Improvement ΔT2–T0** | — | −4.8 ± 2.7 | −3.2 ± 2.6 | 3.05 | 0.003 |

**Note:** a: Significant decrease in within-group scores comparing T1 with T0 (P < 0.05); b: Significant decrease in within-group scores comparing T2 with T0 (P < 0.05); ΔT1–T0, ΔT2–T0: Represent the improvement in scores from T1 and T2 relative to baseline.

**Supplementary Table 4. Multiple Regression Analysis**

| Dependent Variable | Independent Variable | β value | 95% CI | P-value |
| --- | --- | --- | --- | --- |
| **PANSS Total Score Change (ΔT2–T0)** | Whether Received CBT | −6.2 | −9.1 ~ −3.3 | < 0.001 |
|  | Age | −0.2 | −0.4 ~ 0.1 | 0.214 |
|  | Gender (Male = 1) | 1.1 | −0.3 ~ 2.5 | 0.125 |
|  | Disease Duration | −0.3 | −0.7 ~ 0.1 | 0.124 |
|  | Baseline PANSS Total Score | 0.5 | 0.2 ~ 0.8 | 0.003 |
|  | Comorbidity Count | −1.2 | −2.0~−0.4 | 0.002 |
|  | Smoking (Smoker = 1) | -2.0 | -3.5 ~ -0.5 | 0.014 |
|  | Drinking (Drinking = 1) | -1.0 | -2.3 ~ 0.3 | 0.142 |
|  | Social Support (Low = 0, High = 1) | -3.5 | -5.2 ~ -1.2 | 0.001 |
|  | Physical Activity (Low = 0, High = 1) | 2.1 | 0.3 ~ 3.5 | 0.020 |
| **MoCA Score Change (ΔT2–T0)** | Whether Received CBT | 1.3 | 0.6- 2.0 | < 0.001 |
|  | Age | −0.1 | −0.3 ~ 0.1 | 0.384 |
|  | Gender (Male = 1) | 0.2 | −0.4 ~ 0.9 | 0.476 |
|  | Disease Duration | 0.1 | −0.2 ~ 0.4 | 0.351 |
|  | Baseline PANSS Total Score | 0.3 | 0.1 ~ 0.5 | 0.012 |
|  | Comorbidity Count | −0.3 | −0.6 ~ 0.1 | 0.101 |
|  | Smoking (Smoker = 1) | -0.8 | -1.5 ~ -0.2 | 0.031 |
|  | Drinking (Drinking = 1) | 0.2 | -0.5 ~ 1.0 | 0.423 |
|  | Social Support (Low = 0, High = 1) | 1.0 | 0.3 ~ 1.7 | 0.008 |
|  | Physical Activity (Low = 0, High = 1) | 1.5 | 0.7 ~ 2.3 | 0.002 |
| **Adjusted for Depression and Anxiety Scores** | Whether Received CBT | 1.2 | 0.5 ~ 1.9 | < 0.001 |
|  | PHQ-9 | −0.2 | −0.4 ~ 0.1 | 0.132 |
|  | GAD-7 | −0.1 | −0.3 ~ 0.1 | 0.265 |
